# Combined femoral and axillary perfusion strategy for Stanford type a aortic dissection repair

**DOI:** 10.1186/s13019-020-01371-0

**Published:** 2020-11-10

**Authors:** Ling-chen Huang, Qi-chen Xu, Dao-zhong Chen, Xiao-fu Dai, Liang-wan Chen

**Affiliations:** Department of Cardiovascular Surgery, Union Hospital, Fujian Medical University, Fuzhou, 350001 People’s Republic of China

**Keywords:** Aortic dissection, Artery cannulation, Malperfusion

## Abstract

**Background:**

The optimal cannulation strategy in surgery for Stanford type A aortic dissection is critical to patient survival but remains controversial. Different cannulation strategies have their own advantages and drawbacks during cardiopulmonary bypass. Our centre used a combined femoral and axillary perfusion strategy for the surgical treatment of type A aortic dissection. The purpose of this study was to review and clarify the clinical outcome of femoral artery cannulation combined with axillary artery cannulation for the treatment of Stanford type A aortic dissection.

**Methods:**

We performed a retrospective study that included 327 patients who were surgically treated for type A aortic dissection in our institution from January 2017 to June 2019. Femoral and axillary artery cannulation was used to establish cardiopulmonary bypass in patients with type A aortic dissection. The demographic data, surgical data, and clinical results of the patients were calculated.

**Results:**

Femoral artery combined with axillary artery cannulation was technically successful in 327 patients. The cardiopulmonary bypass time was 141.60 ± 34.89 min, and the selective antegrade cerebral perfusion time was 14.94 ± 2.76 min. The early mortality rate was 3.06%. The incidence of permanent neurologic dysfunction was 0.92%. Sixteen patients had postoperative renal insufficiency, and five patients had liver failure.

**Conclusion:**

Femoral artery combined with axillary artery cannulation for type A aortic dissection can significantly improve the prognosis of patients, especially in terms of cerebral protection, and can reduce the occurrence of adverse malperfusion syndrome and neurological complications.

## Introduction

Stanford type A aortic dissection (TAAD) is a surgical emergency with high rates of surgical complications and mortality [1, 2]. The optimal cannulation strategy in surgery for TAAD is critical to patient survival but remains controversial [3–6]. Because the lesion mainly involves the ascending aorta and the aortic arch, the ascending aorta is often not suitable for cannulation to set up the extracorporeal circulation. Various cannulation techniques to establish cardiopulmonary bypass (CPB) for the emergency treatment of TAAD have been reported [7–9]. The femoral artery and the axillary artery have become common alternative cannulation sites. Each of these cannulation sites has its own advantages and disadvantages, and the selection strategy is often affected by the range of lesion involvement, surgical methods, peripheral vascular lesions and other factors.

The femoral artery is the classic cannulation site for this kind of surgery. Femoral artery cannulation can be easily and safely performed before sternal opening. However, many people question the risk of increased mortality, neurological complications, lower limb ischaemia, malperfusion syndrome, embolism and other problems [3, 10].

Axillary artery cannulation can avoid these problems associated with femoral artery cannulation. In recent years, surgeons have begun to use axillary artery cannulation to treat TAAD due to malperfusion with traditional femoral artery cannulation. This type of cannulation provides effective cerebral protection, besides, aortic dissection is rarely involved the axillary artery to ensure the true lumen blood supply to the greatest extent, facilitating the implementation of anterograde selective cerebral perfusion for brain protection. The direction of physiological blood flow reduces malperfusion complications during CPB and allows anterograde cerebral perfusion during circulatory arrest. However, axillary artery cannulation still has some limitations, such as insufficient flow, which may affect the perfusion of organs [11, 12].

We have combined axillary artery cannulation with femoral artery cannulation to overcome the disadvantages of single cannulation. The strategy has been performed at our institution for more than ten years. This study reviewed the mortality and morbidity of TAAD in our hospital from January 2017 to June 2019 and analysed the safety and efficacy of our combined cannulation strategy.

## Materials and methods

### Patients and methods

The medical records, operative records, and discharge summaries of all patients who underwent TAAD surgery with combined cannulation at our institution from January 2017 to June 2019 were reviewed in an electronic medical record system and picture achieving and communication system. The records were reviewed, and cannulation sites, surgical procedures, and overall clinical outcomes were noted. We were particularly concerned about postoperative lower limb ischaemia, stroke, malperfusion, paraplegia, and death - complications that may be related to the cannulation site and retrograde perfusion.

The ethics committee of Union Hospital of Fujian Medical University approved this study protocol. Informed consent was waived due to the retrospective nature of the study.

### Surgical techniques

All patients were placed supine under general anaesthesia. To avoid malperfusion, the following measures are taken to minimize the risk: 1. the peripheral vascular condition was evaluated by computed tomography images to assess whether there was occlusive disease or dissection involvement; and 2. peripheral blood pressure monitoring and oxygen saturation detection were used to evaluate whether there was occlusive disease or dissection involvement. The affected arteries were not cannulated if significant stenosis or dissection were identified in the preoperative examinations.

We preferred right axillary artery combined with right femoral artery cannulation. The surgeons were divided into two groups and started at the same time. During femoral artery cannulation, after a purse-string suture was placed on the exposed femoral artery, a femoral arterial cannula was inserted into the femoral artery by using the modified Seldinger technique. The right femoral artery was usually cannulated if dissection was absent in the right iliofemoral artery. If both sides were dissected, we exercised great care to ensure that our cannula was placed into the true lumen.

If vital signs were stable, another group continued the procedure, and axillary artery exposure for cannulation was obtained through a 4–6 cm incision approximately 2 cm under the subclavian fossa. The fibres of the pectoralis major muscle were bluntly divided. The pectoralis minor muscle was retracted laterally. After exposing the axillary artery, femoral artery clamps were used proximal and distal to the cannulation site. While clamping the vessel and longitudinal arteriotomy, either direct cannulation with an arterial cannula or end-to-side anastomosis with a Dacron graft to the axillary artery with a running 5–0 Prolene suture was carried out. Flow was evaluated through the cannula by blood return, and if flow was sufficient, the cannula was connected to the arterial line and secured to the skin.

Median sternotomy was performed, and venous cannulation was performed with a 2-stage right atrial cannula or with superior and inferior vena cava cannulation in the case of a combined intracardiac operation. The arterial perfusion line was divided into two branches on the operating table, and the “single pump and double tube method” was adopted for management to transfer the arterial perfusion position and protect the cerebral tissue at different stages of the operation.

After cannulation, the cannulation position was confirmed by observing whether there was clear blood return, whether the pump pressure was normal, and whether there was a significant increase in the pump pressure after pumping 50 ~ 100 ml of liquid to eliminate the possibility of insertion into the dissection. The arterial pressure of the radial and dorsal foot arteries as well as transesophageal echocardiography (TEE) and regional cerebral oxygen saturation were routinely monitored by an anaesthesiologist.

After CPB was initiated, we routinely palpated the aorta and compared the pressures of the dorsal foot artery and radial artery, monitored regional cerebral oxygen saturation and evaluated the area ratio of the true lumen and false lumen in the descending aorta with TEE to evaluate whether there was malperfusion. If the patient was suspected of having intraoperative organ malperfusion due to cannulation of the arterial cannula into the false lumen, we changed the location of the arterial cannula.

During surgery, our brain protection methods were deep hypothermia concomitant with selective antegrade cerebral perfusion (SACP). Additionally, neuroprotective drugs were administered, and the head was cooled with a topical ice hat. The axillary artery was rarely dissected, so we could simply place the cannula inside the true lumen. After circulatory arrest was established, we routinely assessed the backflow in the orifice of the arch vessels and monitored regional cerebral oxygen saturation. If the backflow was reduced or the regional cerebral oxygen saturation value was decreased, we placed another cannula inside the orifice of the carotid artery to prevent decreased cerebral perfusion, which would lead to brain dysfunction [13, 14].

If the dissection involved only the ascending aorta, the ascending aorta and a hemi-arch replacement were routine used. For patients with aortic arch involvement, we usually used triple-branched stent-graft technology [15]. During the cooling phase of core body temperature, the innominate artery and the left common carotid artery were dissociated from the surrounding tissues. After the ascending aorta was clamped, aortic root manipulations such as aortic valve repair and aortic sinus reconstruction were performed, and then the Dacron tube graft was subsequently continuously anastomosed to the aortic root. When a 22 °C rectal temperature was achieved, selective cerebral perfusion via the right axillary artery was started at a rate of 10 to 15 mL/kg/min, and femoral artery perfusion was discontinued. With the innominate artery and left common carotid artery cross-clamped, the ascending aorta was transected at the base of the innominate artery. Then, the triple-branched stent graft was inserted and properly positioned. Finally, continuous end-to-end anastomosis was performed between the artificial vessels and intraoperative stents. After the dissection operation and after the air was carefully flushed out, systemic perfusion was resumed, and the patient was rewarmed [16–18].

### Definition of clinical parameters

Early mortality was defined as all-cause mortality either in-hospital or within 30 days of surgery. Temporary neurologic dysfunction was defined as the presence of postoperative agitation, transient delirium, and confusion without focal neurologic symptoms. Permanent neurologic injury was defined as the new onset of permanent neurologic deficits, including coma or stoke after surgical repair with or without morphological correlates in a brain magnetic resonance or computed tomography image. Early stroke was defined as evident permanent neurologic injury after emergence from anaesthesia. Delayed stroke was defined as permanent neurologic injury after first awakening from surgery without neurological deficits. Acute kidney injury was defined as serum creatinine concentrations over 133 μmol/l or the need for dialysis due to oliguria. Postoperative liver failure was defined as the concurrent observation of at least two of the following parameters: coagulation abnormalities, total bilirubin > 15 mg/dL, liver enzyme levels more than tenfold the upper limit of normal, alteration of consciousness, and asterixis.

### Statistical analysis

Statistical analysis was performed with SPSS ver. 22.0 (SPSS Inc., Chicago, IL, USA). Nonnormally distributed data were expressed as medians. Continuous variables with a normal distribution were expressed as the mean ± standard distribution.

## Results

A total of 327 patients underwent surgical repair for TAAD in our institution between January 2017 and June 2019. There were 253 males and 74 females with a mean age of 52.16 ± 11.72 years (range, 22 to 87 years). There were 261 patients (79.82%) with hypertension and 28 patients (8.56%) with diabetes. The mean BMI was 24.21 ± 1.92 kg/m^2^.

The location of the primary intimal tear was the ascending aorta in 219 patients (66.97%), the arch in 40 (12.23%), and the distal end of the left subclavian artery in 37 (11.31%); no obvious intimal tear was found in 31 (9.48%) patients. The false lumen involved the innominate artery in 49 patients (14.98%), the left common carotid artery in 37 patients (11.31%), the left subclavian artery in 31 patients (9.48%), the celiac trunk in 113 patients (34.56%), the superior mesenteric artery in 60 patients (18.35%), the right renal artery in 90 patients (27.52%), and the left renal artery in 105 patients (32.11%). These lesions were diagnosed on preoperative computed tomography scans and were partly confirmed at surgery.

The ejection fraction was 62.76 ± 7.63%. Echocardiography showed 19 cases of moderate pericardial effusion (5.81%). Massive pericardial effusion that led to pericardial tamponade was noted in 5 patients (1.53%). There were 170 patients (51.99%) with mild aortic insufficiency, 116 patients (35.47%) with moderate aortic insufficiency, and 41 patients (12.54%) with severe aortic insufficiency. Coronary artery involvement was observed in a total of 11 patients (3.36%) before surgery, and regional wall motion abnormalities were identified in 17 patients (5.20%) with echocardiography.

In seven patients (2.14%), preoperative neurologic symptoms such as transient ischaemic attack and transient drowsy mentality developed. Eleven patients had preoperative renal insufficiency, and four patients had hepatic insufficiency. Two patients suffered from paraplegia. The clinical characteristics of the patients are detailed in Table 1.

**Table 1:** Demographic and clinical data

Item	Data
Male/Female	253/74
Age (years)	52.16 ± 11.72
BMI (kg/m^2^)	24.21 ± 1.92
Hypertension (*n*, %)	261 (79.82%)
Diabetes (*n*, %)	28 (8.56%)
LVEF (%)	62.76 ± 7.63%
location of intimal tear (*n*, %)	
ascending aorta	219 (66.97%)
aortic arch	40 (12.23%)
descending aorta	37 (11.31%)
without obvious intimal tear	31 (9.48%)
Artery involved by the false lumen (*n*, %)	
Innominate artery	49 (14.98%)
left common carotid artery	37 (11.31%)
the left subclavian artery	31 (9.48%)
Celiac trunk artery	113 (34.56%)
superior mesenteric artery	60 (18.35%)
right renal artery	90 (27.52%)
left renal artery	105 (32.11%)
Pericardial tamponade (*n*, %)	5 (1.53%)
Aortic insufficiency (*n*, %)	
mild	170 (51.99%)
moderate	116 (35.47%)
severe	41 (12.54%)
Observed Coronary artery involved (*n*, %)	11 (3.36%)
Regional wall motion abnormalities (*n*, %)	17 (5.20%)
Neurologic Symptoms (*n*, %)	7 (2.14%)
Cardiac tamponade (*n*, %)	13 (3.98%)
Renal insufficiency (*n*, %)	11 (2.36%)
Hepatic insufficiency (*n*, %)	4 (1.22%)
Paraplegia (*n*, %)	2 (0.61%)
Transient leg ischemia (*n*, %)	3 (0.92%)

The ascending aorta was replaced in all cases. The most frequent extent of aortic replacement was the ascending aorta and hemiarch replacement combined with modified triple-branched stent-graft implantation for the repair of TAAD. The detailed operative procedures are shown in Table 2. For proximal repair, supracoronary anastomosis was performed in 120 patients (36.70%), root reconstruction was performed in 171 patients (55.35%) in whom the aortic valve could be preserved, and aortic valve replacement was performed in 36 patients (11.00%). A coronary artery bypass graft was performed in 11 (3.36%) patients.

**Table 2:** Intra-operative data

Item	Data
Surgery strategy	
Hemiarch replacement (*n*, %)	16 (4.89%)
Modified triple-branched implantation (*n*, %)	311 (95.11%)
Proximal repair	
Supracoronary anastomosis alone	120 (36.70%)
Aortic valve replacement	36 (11.00%)
Root reconstruction	171 (55.35%)
Coronary artery bypass graft	11 (3.36%)
Cardiopulmonary bypass time (min)	141.60 ± 34.89
Cross-clamping time (min)	49.05 ± 20.16
Selective cerebral perfusion time (min)	14.94 ± 2.76
Cerebral perfusion strategy	
Unilateral (*n*, %)	65 (19.88%)
Bilateral (*n*, %)	262 (80.12%)

The CPB time was 90 to 334 min (mean, 141.60 ± 34.89 min), the cross-clamp time was 22 to 169 min (mean, 49.05 ± 20.16 min), and the selective cerebral perfusion and lower body arrest time was 7 to 25 min (mean, 14.94 ± 2.76 min). Sixty-five patients had unilateral selective cerebral perfusion, and 262 had bilateral selective cerebral perfusion.

The postoperative consciousness time and mechanical ventilation time after the operation were 6.71 ± 2.35 and 17.29 ± 2.10 h, respectively. The postoperative length of stay in the intensive care unit was 55.41 ± 10.16 h. No patient died during the operation. There were 10 (3.06%) early deaths. Three patients died of sepsis on postoperative days 7, 21, and 23. The other five patients died of multiorgan failure. One patient had a low postoperative cardiac output with extracorporeal membrane oxygenation (ECMO) support and died on postoperative day 5. Another patient died of haemorrhagic stroke on postoperative day 30. Eight patients were treated surgically but were discharged from hospital before fully recovery and were lost to follow-up. Transient neurologic dysfunction was observed in 15 patients (4.59%), and all patients recovered before hospital discharge. A total of 3 patients (0.92%) suffered from permanent neurologic dysfunction. Haemorrhagic and ischaemic stroke was observed in one patient and two patients, respectively. Two patients with preoperative paraplegia also had paraplegia postoperatively. Five patients with preoperative cerebral malperfusion recovered without postoperative neurologic deficits or sequelae. Sixteen patients experienced postoperative haemodialysis-dependent renal failure (4.89%). Five cases of postoperative acute liver failure were documented (1.53%). No patients required repeat surgery to correct excessive postprocedural bleeding. Lower limb ischaemia occurred in 3 patients (0.92%). No patients had permanent cannulation-related complications. All related postoperative complications are shown in Table 3.

**Table 3:** Postoperative Data

Item	TA group
Early Mortality	10 (3.06%)
Postoperative consciousness time (hours)	6.71 ± 2.35
Mechanical ventilation time (hours)	17.29 ± 2.10
ICU stay (hours)	55.41 ± 10.16
Transient neurologic dysfunction (*n*, %)	15 (4.59%)
Permanent neurologic dysfunction (*n*, %)	3 (0.92%)
Hemodialysis-dependent renal failure (*n*, %)	16 (4.89%)
Liver failure (*n*, %)	5 (1.53%)
Lower limb ischemia (*n*, %)	3 (0.92%)

## Discussion

The main finding of our study was that the early clinical outcome of patients undergoing surgical repair for type A aortic dissection with a combined femoral and axillary perfusion strategy in our institution was similar with that of patients with other cannulation strategy. Our 30-day mortality rate and other adverse complications are similar to associations reported in other studies [17–21].

Surgical repair of type A aortic dissection is associated with high mortality and high complication morbidity. Establishing CPB that maintains adequate systemic perfusion is essential to prevent end-organ hypoperfusion during the procedure. Femoral artery cannulation and axillary artery cannulation to establish extracorporeal circulation are widely used in clinical practice and have been extensively reported in previous literature [22–24].

The femoral artery has been used as the primary cannulation site for CPB in cardiac surgery for more than 40 years [25, 26]. Several studies have shown that retrograde perfusion of the femoral artery has the following deficiencies: (1) a “live valve” effect and false lumen blood supply, leading to central nervous system, upper arm, kidney and abdominal organ perfusion disorders; (2) a false lumen blood supply and retrograde blood flow flush atherosclerotic plaques on the vascular wall, leading to embolization and an increase in the incidence of stroke; (3) compared with that of the axillary artery, femoral artery cannulation is easily affected by atherosclerosis or dissection; (4) lower limb ischaemia; and (5) the inconvenience of anterograde selective cerebral perfusion [27, 28].

Since 1995, axillary artery cannulation has been used as an alternative to femoral artery cannulation in patients with TAAD [29]. Compared with femoral artery cannulation, axillary artery cannulation can significantly reduce central nervous system complications in patients with deep hypothermia. Anterograde selective cerebral perfusion is considered to be the most ideal method for cerebral protection at present, and axillary artery cannulation can be very convenient for coordinating brain protection, which greatly reduces the side effects resulting from deep hypothermia and circulatory arrest (DHCA). Yoshimasa Moizumi believes that antegrade perfusion through the axillary artery can avoid the disadvantages of retrograde perfusion, such as organ malperfusion caused by floating dissected intima flaps or expanding false lumens. In his study, 69 patients (65.09%) underwent cannulation of the axillary artery, and the results demonstrated decreased hospital mortality [30, 31]. In addition, the advantages of axillary artery cannulation are also shown in the following aspects. 1. There is rich collateral circulation of the neck and shoulder. A Chinese study showed that 17% of patients have an incomplete anterior and posterior circle of Willis. A complete circle of Willis configuration was only found in 27% of the Chinese population, and 56% had a partially complete circle of Willis [32]. 2. Axillary artery cannulation is not easily affected by atherosclerosis or dissection. 3. Axillary artery cannulation avoids retrograde embolism and expansion of the dissection. 4. Axillary artery cannulation facilitates selective cerebral perfusion.

One disadvantage of axillary artery cannulation is that it takes more time to construct. However, Budde et al. used axillary artery cannulation to establish CPB in 61 patients undergoing elective and emergency surgery due to proximal aortic pathology. There were no significant differences in postoperative neurological dysfunction or mortality between the selective group and the emergency group. They claimed that the routine use of axillary artery cannulation in emergent cases was just as safe and efficacious as its use in the elective setting [33]. However, the biggest disadvantage of axillary artery cannulation is insufficient flow, which may affect the perfusion of organs [12].

Our centre used femoral artery combined with axillary artery cannulation to establish extracorporeal circulation, providing reliable circulation support for TAAD repair. Few studies have examined the clinical outcomes of this cannulation strategy. In this study, 96.94% (317/327) of the patients undergoing femoral and axillary artery cannulation survived. In addition, the incidences of permanent neurologic dysfunction, renal insufficiency, liver failure and lower limb ischaemia were 0.92, 4.89, 1.53 and 0.92%, respectively. Based on the results of our institution, we conclude that femoral artery combined with axillary artery cannulation results in satisfactory short-term outcomes.

With the combination of femoral artery and axillary artery cannulation, with this single pump and double tube method, the true lumen of TAAD can be fully perfused to the greatest extent during CPB, avoiding poor systemic perfusion. In addition, anterograde cerebral perfusion can also be carried out through the upper body during circulatory arrest, which greatly ensures cerebral perfusion and reduces the incidence of cerebral complications [34].

## Conclusion

Femoral artery combined with axillary artery cannulation for Stanford type A AD can significantly improve the prognosis of patients, especially in terms of cerebral protection, and can reduce the occurrence of adverse malperfusion syndrome.

## Data Availability

Data sharing was not applicable to this article, as no data sets were generated or analysed during the current study.

## Abbreviations

TAAD: Type A Aortic dissection
TEE: Transesophageal echocardiography
CPB: Cardiopulmonary bypass
SACP: Selective antegrade cerebral perfusion
DHCA: Deep hypothermia and circulatory arrest
ECMO: Extracorporeal membrane oxygenation
